# Exploration of key stress granule-related genes in chronic sinusitis based on transcriptomics

**DOI:** 10.1038/s41598-026-57646-4

**Published:** 2026-06-18

**Authors:** Luping Zhu, Mingzhe Yang, Yuan Chen, Shu Zhang, Xiaoxue Wang, Kaile Zhang, Xiaobing Sun

**Affiliations:** 1 Department of Otorhinolaryngology, The Second Affiliated Hospital of Nanjing Medical University, 121 Jiangjiayuan Road, Gulou District, Nanjing, 210011 China; 2 Nanjing Medical University, Nanjing, China

**Keywords:** Chronic rhinosinusitis with nasal polyps, Stress granules, Key genes, Machine learning, Immune infiltration analysis, Biomarkers, Computational biology and bioinformatics, Diseases, Genetics, Immunology

## Abstract

Chronic rhinosinusitis with nasal polyps (CRSwNP) frequently induces the formation of intracellular stress granules (SGs). This study aims to identify key genes linked to both CRSwNP and SGs, offering novel perspectives for improving CRSwNP treatment and management. Data from GSE136825, GSE179265, and SG-related gene sets were retrieved from public databases. Key genes were identified via machine learning, ROC analysis, and expression validation. Subsequently, nomogram construction, Gene Set Enrichment Analysis (GSEA), immune infiltration analysis, and drug prediction were performed to explore the regulatory mechanisms of these genes. Three candidate genes—ANG, CRYAB, and FBP1—passed ROC and expression validation and were ultimately identified as key players. GSEA revealed that these genes collectively participate in the cell cycle pathway. Immune infiltration analysis showed 23 differentially abundant immune cell types between the CRSwNP and control groups, with ANG, CRYAB, and FBP1 all exhibiting significant positive correlations with Type 2 T helper cells. Furthermore, estradiol, copper sulfate, and trichostatin A were identified as drugs interacting with all three key genes. These findings provide valuable insights for advancing CRSwNP diagnosis.

**Supplementary Information:**

The online version contains supplementary material available at https://doi.org/10.1038/s41598-026-57646-4.

## Introduction

Chronic rhinosinusitis with nasal polyps (CRSwNP) is a heterogeneous condition characterized by persistent inflammation of the nasal and sinus mucosa lasting over 12 weeks, accompanied by the formation of nasal polyps^1^. Patients with CRSwNP experience severe symptoms such as nasal obstruction, discharge or posterior rhinorrhea, anosmia, and facial pressure or headache. Affecting approximately 5% of the global population, CRSwNP significantly impacts patients’ quality of life and imposes a considerable medical burden^2^. Despite the continuous use of nasal corticosteroid sprays or surgical intervention, CRSwNP is prone to recurrence. In 20–45% of cases, CRS is associated with comorbidities such as bronchial asthma, and a markedly elevated risk of COPD, depression, or obstructive sleep apnea^3^. The disease may progress to more severe phenotypes, including aspirin-exacerbated respiratory disease (AERD)^4^. Its etiology involves a complex interaction between genetic factors (e.g., IL-4R and IL-5 gene polymorphisms), environmental triggers (pollutants, allergens), microbial dysbiosis, and immune dysfunction^5^. CRSwNP can be categorized into distinct endotypes based on factors such as inflammation markers, infiltrating cell types, tissue remodeling, and comorbid conditions^6^. The condition is marked by pronounced eosinophilic infiltration and a type 2 inflammatory response. However, the heterogeneous nature of CRSwNP has obscured the understanding of its pathogenic mechanisms and hindered the identification of precise biomarkers. Thus, there is an urgent need to explore the molecular mechanisms driving CRSwNP, identify key genes, and establish a scientific foundation for early intervention and personalized treatment.

Stress granules (SGs) are cytoplasmic condensates composed of RNA and proteins that form specifically under stress conditions. SGs temporarily sequester mRNAs and proteins, protecting them from autophagic and proteasomal degradation, allowing cells to resume translation and activate other signaling pathways upon stress recovery^7^. The formation of SGs is a highly conserved mechanism aimed at minimizing cellular damage in adverse environments^8^, such as nutrient deprivation, heat, and oxidative or osmotic stress^9^. SGs play a dual role in neurodegenerative diseases, either supporting cellular adaptation to stress through protective mechanisms or contributing to pathological dysfunction^10^. Abnormal SG assembly is linked to cellular dysfunction in various pathological conditions, including cardiovascular disease, cancer, viral^11^ and bacterial infections^12^, and degenerative disorders. During infection, SGs are induced and modulate immune cell activity, enhancing the cellular defense against pathogens. However, their role varies between viral and microbial infections and depends on the immune cells involved^12^. While current technologies have facilitated the identification of CRSwNP biomarkers, the relationship between CRSwNP and SGs, as well as the underlying molecular mechanisms, remains unclear.

This study aims to systematically identify SG-associated biomarkers through bioinformatics methods using publicly available transcriptomic datasets, exploring their regulatory roles in CRSwNP. The findings are expected to uncover potential therapeutic targets for CRSwNP diagnosis and treatment. To achieve this, weighted gene co-expression network analysis (WGCNA) was applied to the GSE136825 and GSE179265 datasets to identify key genes associated with both SGs and CRSwNP. Additionally, bioinformatics techniques such as immune infiltration analysis and drug prediction were employed to investigate key biological pathways and their molecular regulatory mechanisms. Through this analysis, this study aims to elucidate the genetic and molecular mechanisms underlying CRSwNP development and progression in relation to SGs, offering novel insights into the role of SGs in CRSwNP.

## Results

### There were 23 candidate genes linked to SGs

**Fig. 1:**
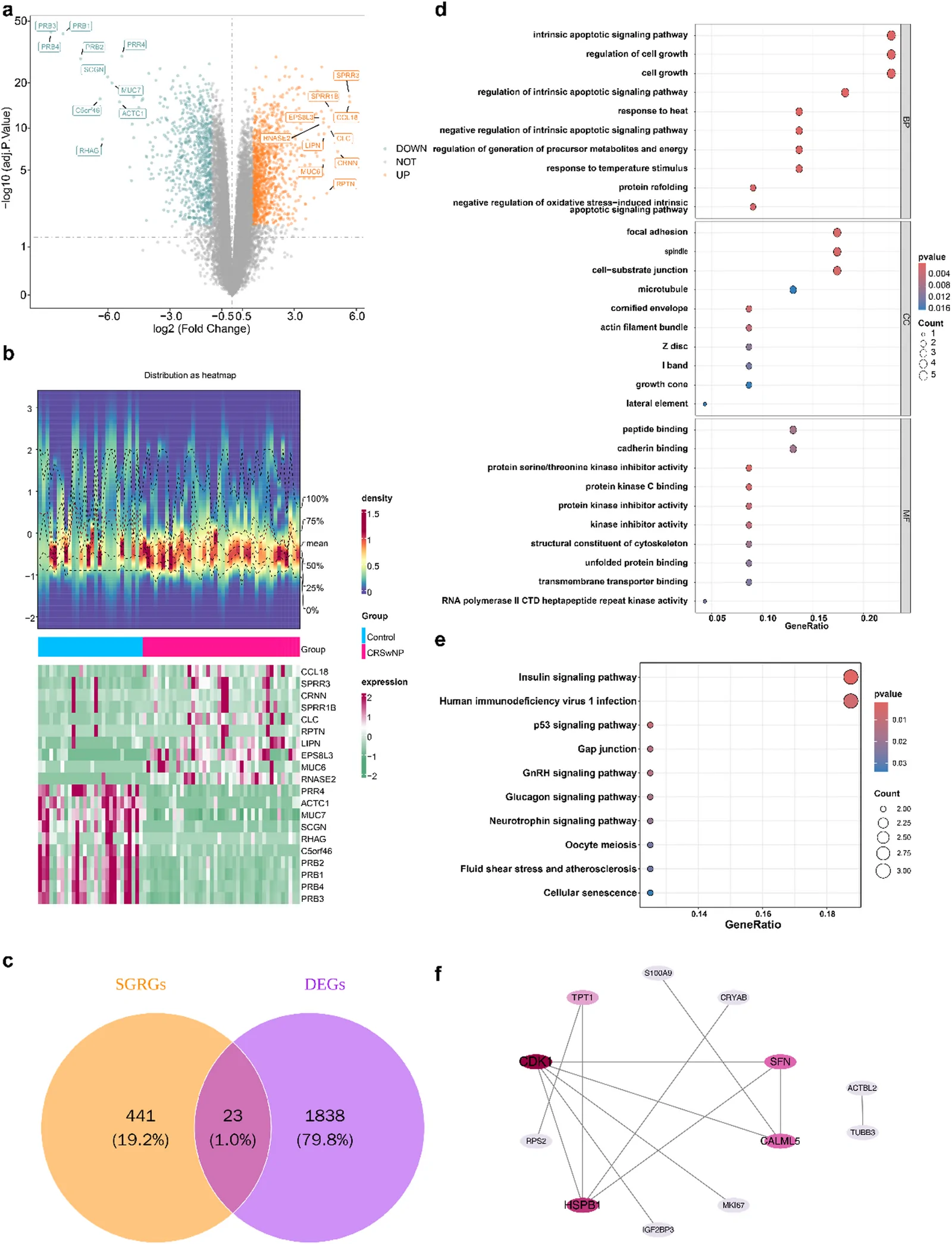
Identification of candidate genes. (**a**) Volcano plot of DEGs. (**b**) Heatmap of DEGs. (**c**) Venn diagram for candidate gene screening (DESGRGs). (**d**) Enrichment analysis of candidate genes. (**e**) Top pathways from KEGG enrichment analysis.The Kyoto Encyclopedia of Genes and Genomes (KEGG) pathway map was obtained from KEGG(https://www.kegg.jp/). KEGG is a publicly available resource under the terms of the academic uselicense^13–15^. (**f**) Interaction relationships among the candidate genes.

A total of 1861 differentially expressed genes (DEGs) were identified between the CRSwNP and control samples in the training set, including 1102 upregulated and 759 downregulated genes (Fig. 1a, b, Supplementary Table 1). To further pinpoint DEGs associated with SGs, the 1861 DEGs were intersected with 464 SG-related genes, yielding 23 candidate genes (Fig. 1c, Supplementary Table 2). Gene Ontology (GO) enrichment analysis was performed on these candidate genes (*P* < 0.05), resulting in 300 pathways, of which five are presented here (Fig. 1d, Supplementary Table 3). Biological processes (BP) included the intrinsic apoptotic signaling pathway, cellular components (CC) included focal adhesion, and molecular functions (MF) included peptide binding. Additionally, Kyoto Encyclopedia of Genes and Genomes (KEGG) pathway analysis revealed the top 89 pathways, with osteoclast differentiation and insulin signaling being among the most prominent (Fig. 1e, Supplementary Table 4). It should be noted that, since enrichment analysis is an exploratory method, we used uncorrected p-values (*p* < 0.05) and did not perform FDR correction. Therefore, the results may have a risk of false positives and require further validation. The protein–protein interaction (PPI) network analysis identified 11 isolated target genes among the 23 candidates, with 12 nodes and 12 edges, suggesting 12 interaction relationships among the genes (Fig. 1f). This comprehensive analysis offers valuable insights for further research into the underlying mechanisms of CRSwNP and the development of innovative therapeutic strategies.

### ANG, CRYAB, and FBP1 showed an excellent diagnostic ability for CRSwNP

**Fig. 2:**
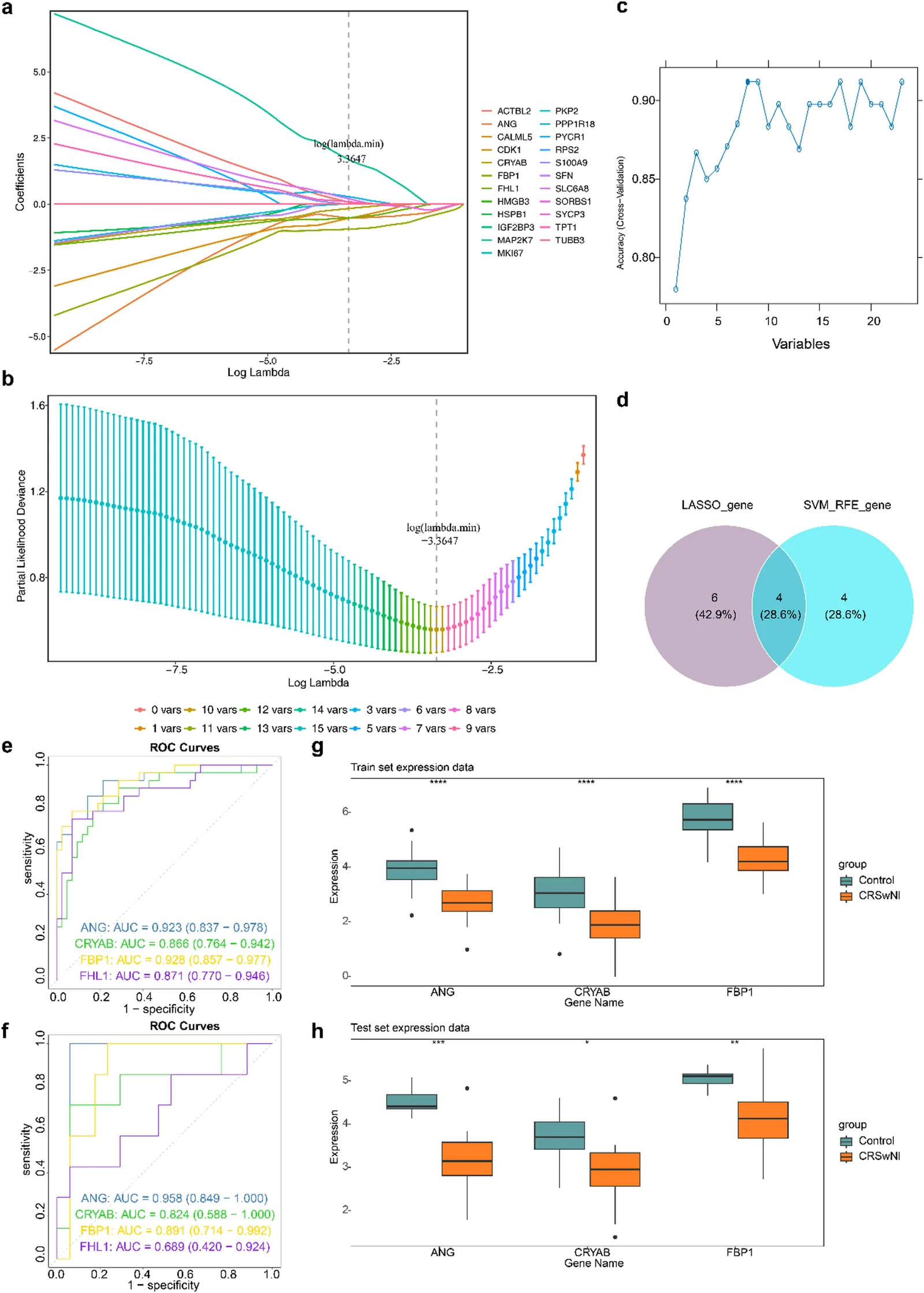
Identification of characteristic genes. (**a**) LASSO regression analysis for gene selection. **(b**) Generalization error *versus* number of features. (**c**) SVM–RFE analysis results. (**d**) Machine learning-based identification of key genes. (**e**) Diagnostic value assessment of key genes. (**f**) Diagnostic value assessment of key genes. (**g**) Biomarker expression in the GSE136825 training set. (**h**) Biomarker expression in the GSE179265 test set.

Subsequently, a feature gene set was identified using a model with the lowest error rate, resulting in ten genes when lambda.min = − 3.3647: ACTBL2, ANG, CALML5, CRYAB, FBP1, FHL1, MAP2K7, PPP1R18, SLC6A8, and TPT1 (Fig. 2a, b). Additionally, the SVM–RFE model achieved optimal performance with 8 variables, and further increasing the number of variables did not enhance the accuracy. This model identified 8 genes as feature gene set 2, including FBP1, ANG, SORBS1, PYCR1, SYCP3, TUBB3, FHL1, and CRYAB (Fig. 2c). Notably, the AUC values for both machine learning models exceeded 0.9 (Supplementary Fig. 1). Finally, four genes (ANG, CRYAB, FBP1, and FHL1) were identified as intersecting candidates from both algorithms (Fig. 2d).

The ROC analysis of the four candidate genes revealed that only three genes—ANG, CRYAB, and FBP1—had AUC values greater than 0.7 in both the training and validation sets. Therefore, these three genes were selected as key candidates (Fig. 2e, f). Furthermore, significant expression differences (*P* < 0.05) were observed for these genes, with consistent expression trends between CRSwNP and control samples in both the GSE136825 and GSE179265 datasets (Fig. 2g, h). ANG, CRYAB, and FBP1 were thus selected as key genes for further investigation.

### ANG, CRYAB, and FBP1 have strong diagnostic capabilities and are located on different chromosomes

**Fig. 3:**
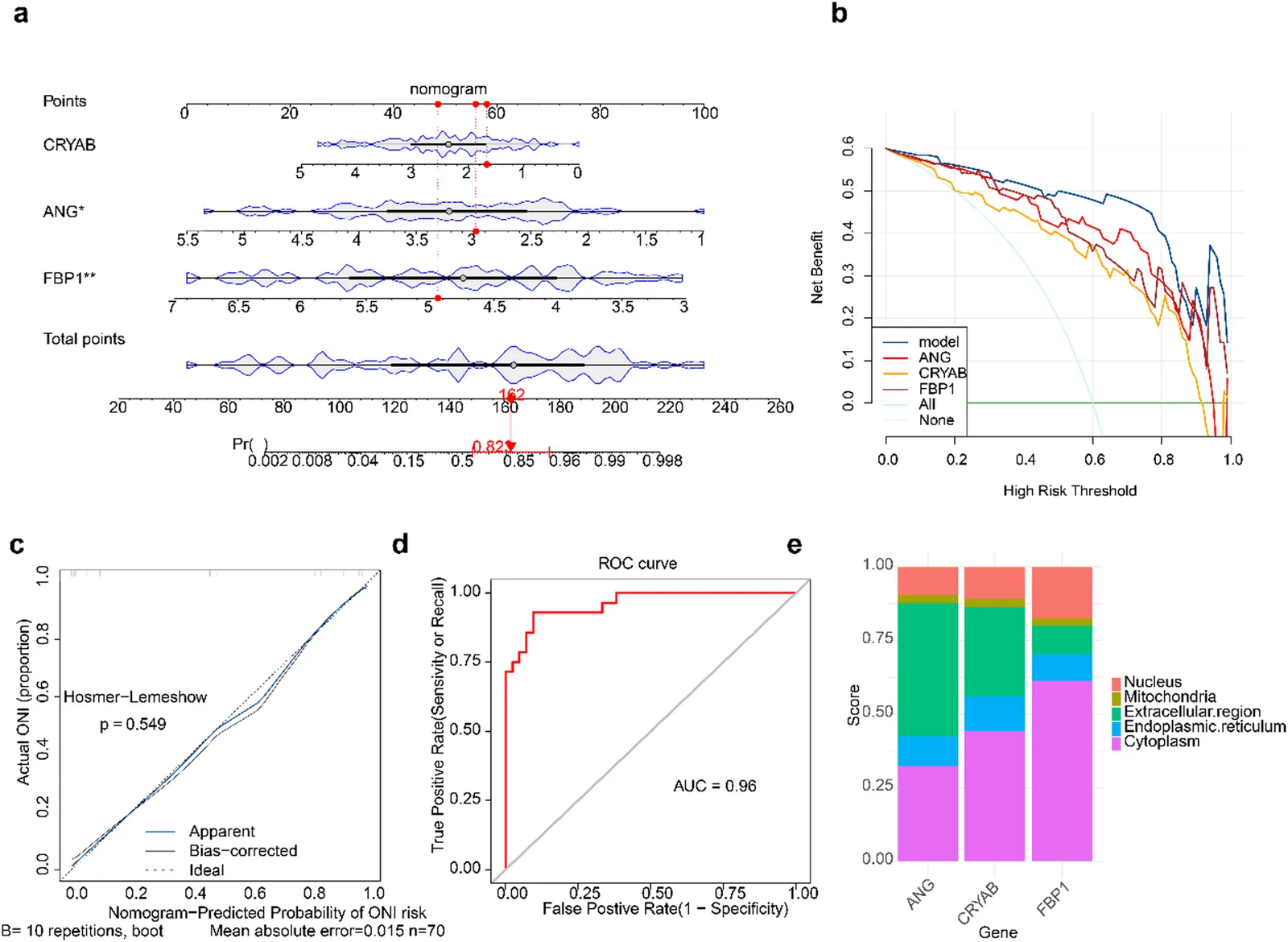
Construction of nomogram. (**a**) Nomogram based on biomarker construction. (**b**) Decision curve analysis (DCA) comparing the nomogram with individual genes for clinical prediction. (**c**) Calibration curves assessing the predictive accuracy of the nomogram model. (**d**) Receiver operating characteristic (ROC) curve of the nomogram, demonstrating excellent discriminative ability (AUC = 0.96). (**e**) Subcellular localization analysis map of biomarkers. The horizontal axis represents the three genes: ANG, CRYAB, and FBP1. The vertical axis shows the score. Colors represent different compartments: pinkish–purple for cytoplasm, blue for endoplasmic reticulum, teal for extracellular region, olive green for mitochondria, and coral red for the nucleus.

The nomogram analysis revealed a prediction probability of approximately 0.82 (Fig. 3a). Decision curve analysis (DCA) demonstrated that the nomogram curve consistently outperformed individual gene curves, indicating the reliability and clinical applicability of the nomogram model (Fig. 3b). The calibration curve exhibited a p-value of 0.549 (well above 0.05), suggesting no significant discrepancy between the predicted and observed values. Additionally, a mean absolute error (MAE) of less than 0.1 indicated minimal deviation between the predicted and actual disease risk, further emphasizing the high precision of the nomogram model for CRSwNP prediction (Fig. 3c). The ROC curve analysis revealed that the AUC values of the nomogram were greater than 0.7 in both the training set and the validation set (Fig. 3d, Supplementary Fig. 2). These results suggest that the constructed nomogram demonstrates excellent discriminative ability, effectively distinguishing between positive and negative cases (diseased and non-diseased individuals). However, due to the limited sample size, there is a potential risk of overfitting, necessitating further validation in larger prospective cohorts.

Subcellular localization analysis revealed that FBP1 is predominantly localized in the cytoplasm (~ 60%), with lesser concentrations in the extracellular region, endoplasmic reticulum, and nucleus. CRYAB is mainly distributed in the cytoplasm (~ 45%), extracellular region (~ 30%), and nucleus (~ 17%), totaling over 90% of its localization. ANG is primarily found in the extracellular region (~ 45%) and cytoplasm (~ 35%), with negligible presence in the other compartments (Fig. 3e).

### Construction of molecular regulatory network

**Fig. 4:**
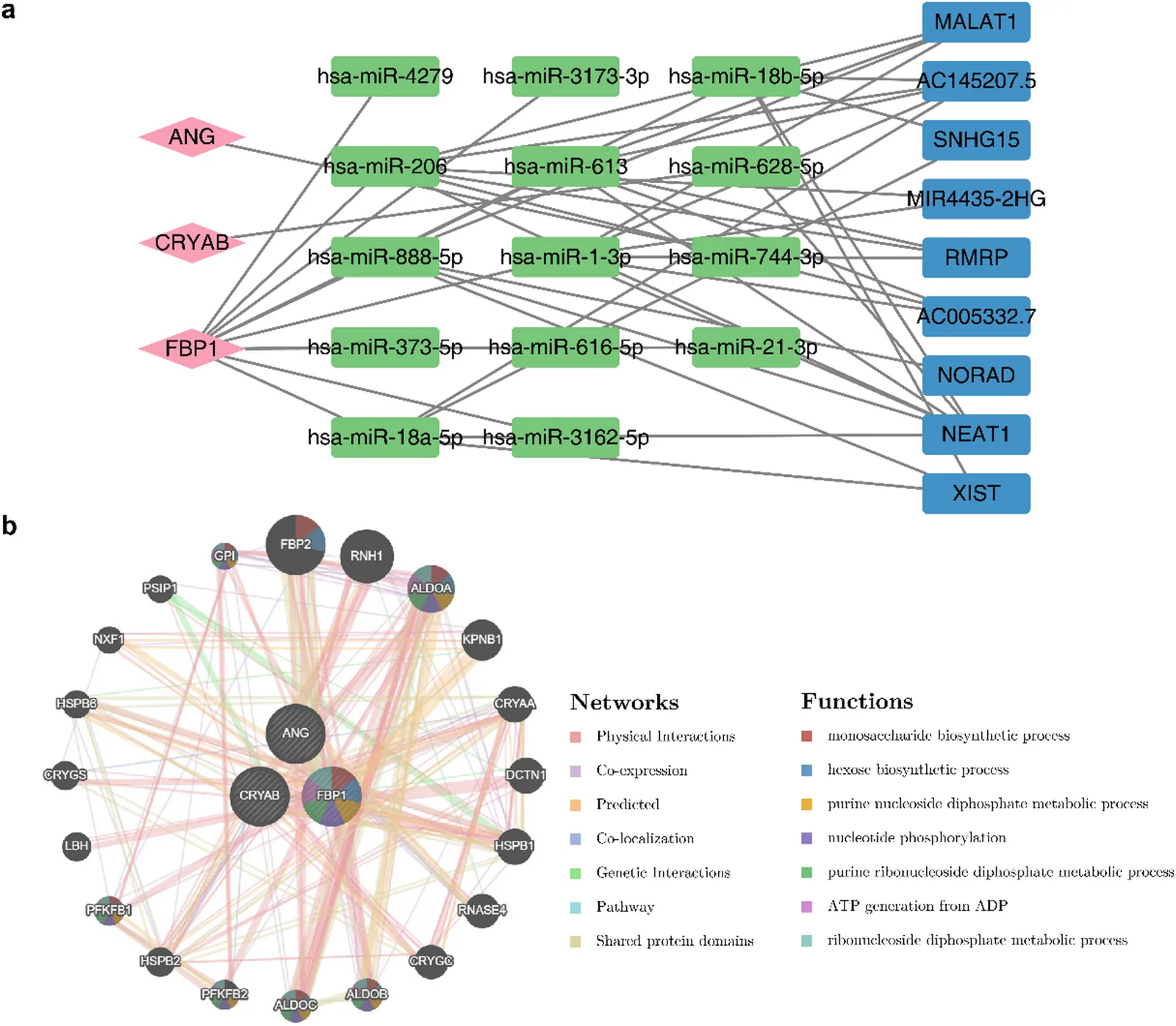
Construction of molecular regulatory networks. (**a**) Diagram of the mRNA–miRNA–lncRNA regulatory network, with pink representing biomarkers, green representing miRNAs, and blue representing lncRNAs. (**b**) Gene–gene interaction network of key genes analyzed using the GeneMANIA database, showing the 20 most frequently interacting genes.

The molecular regulatory network analysis indicated the following: pink nodes represent the three key genes, while green nodes correspond to miRNAs (14 predicted), and blue nodes represent lncRNAs (9 predicted) (Fig. 4a). GeneMANIA analysis identified 20 genes interacting with the key genes ANG, CRYAB, and FBP1, including FBP2, RNH1, ALDOA, KPNB1, CRYAA, DCTN1, HSPB1, RNASE4, CRYGC, ALDOB, ALDOC, PFKFB2, HSPB2, PFKFB1, LBH, CRYGS, HSPB6, NXF1, PSIP1, and GPI. These genes are primarily involved in biological processes such as fructose-bisphosphatase 2 activity, ribonuclease/angiogenin inhibitor 1 function, aldolase activity in fructose-bisphosphate A metabolism, karyopherin subunit beta 1-mediated transport, and crystallin alpha A-related protein structure maintenance (Fig. 4b).

### Immune infiltration analysis of key genes and enrichment analysis

**Fig. 5:**
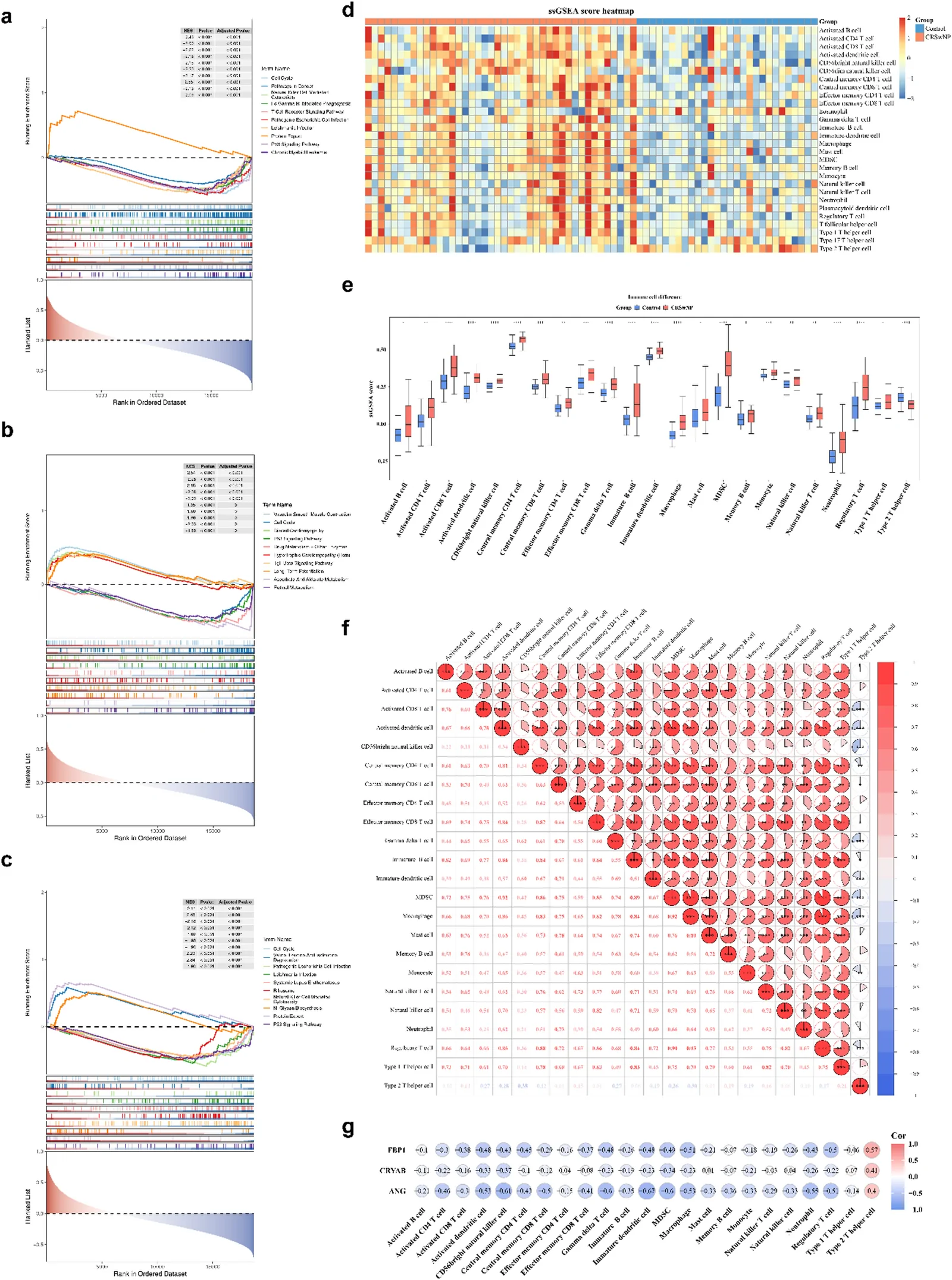
Enrichment analysis of GSEA. (**a**) GSEA of ANG. (**b**) GSEA of CRYAB. (**c**) GSEA of FBP1. (**d**) Comparison of immune cell infiltration abundance between the CRSwNP and control groups. (**e**) Comparison of differential immune cell infiltration between the CRSwNP and control groups. (**f**) Correlation analysis among 23 types of infiltrating immune cells (× indicates no correlation, red indicates a positive correlation, blue indicates a negative correlation, and the value represents the degree of correlation). (**g**) Correlation analysis between key genes and differentially infiltrated immune cells, demonstrating that ANG, CRYAB, and FBP1 exhibit significant positive correlations with type 2 T helper cells, with negative or no correlations with other immune cell types

Gene Set Enrichment Analysis (GSEA) results indicated that ANG was enriched in 68 pathways, CRYAB in 34 pathways, and FBP1 in 50 pathways. The cell cycle pathway was the most significantly shared pathway among the three key genes (Fig. 5a–c, Supplementary Tables 5–7).

Immune cell infiltration analysis revealed significant differences in the infiltration levels of 22 immune cell types between the CRSwNP and control groups. Notably, T cells and natural killer (NK) cells exhibited opposing expression trends (Fig. 5d). A total of 23 immune cell types with differential infiltration were identified, including activated CD4^+^ T cells and activated CD8^+^ T cells (Fig. 5e). Correlation analysis of these infiltrating immune cell types showed that most cell types exhibited positive correlations, with the strongest correlation observed between regulatory T cells and macrophages (cor = 0.93, *P* < 0.05). Within the adaptive immune cells, memory B cells demonstrated a significant positive correlation with central memory CD4⁺ T cells (cor = 0.84, *P* < 0.05). In the immunosuppressive cell populations, myeloid-derived suppressor cells showed a significant positive correlation with regulatory T cells (cor = 0.90, *P* < 0.05). Among the innate immune cells, NK cells exhibited a significant positive correlation with mast cells (cor = 0.65, *P* < 0.05). Notably, type 2 helper T cells showed a negative correlation with most of the differentially infiltrated immune cells (Fig. 5f). Correlation analysis between the key genes and differentially infiltrated immune cells revealed that ANG (cor = 0.40, *P* < 0.05), CRYAB (cor = 0.41, *P* < 0.05), and FBP1 (cor = 0.57, *P* < 0.05) were significantly positively correlated with type 2 T helper cells. In contrast, these genes showed negative or no correlations with the other 23 differentially infiltrated immune cell types (Fig. 5g).

### **Estradiol**,** copper sulfate**,** and trichostatin A were predicted for the three key genes**,** among which estradiol showed strong binding affinity**

**Fig. 6:**
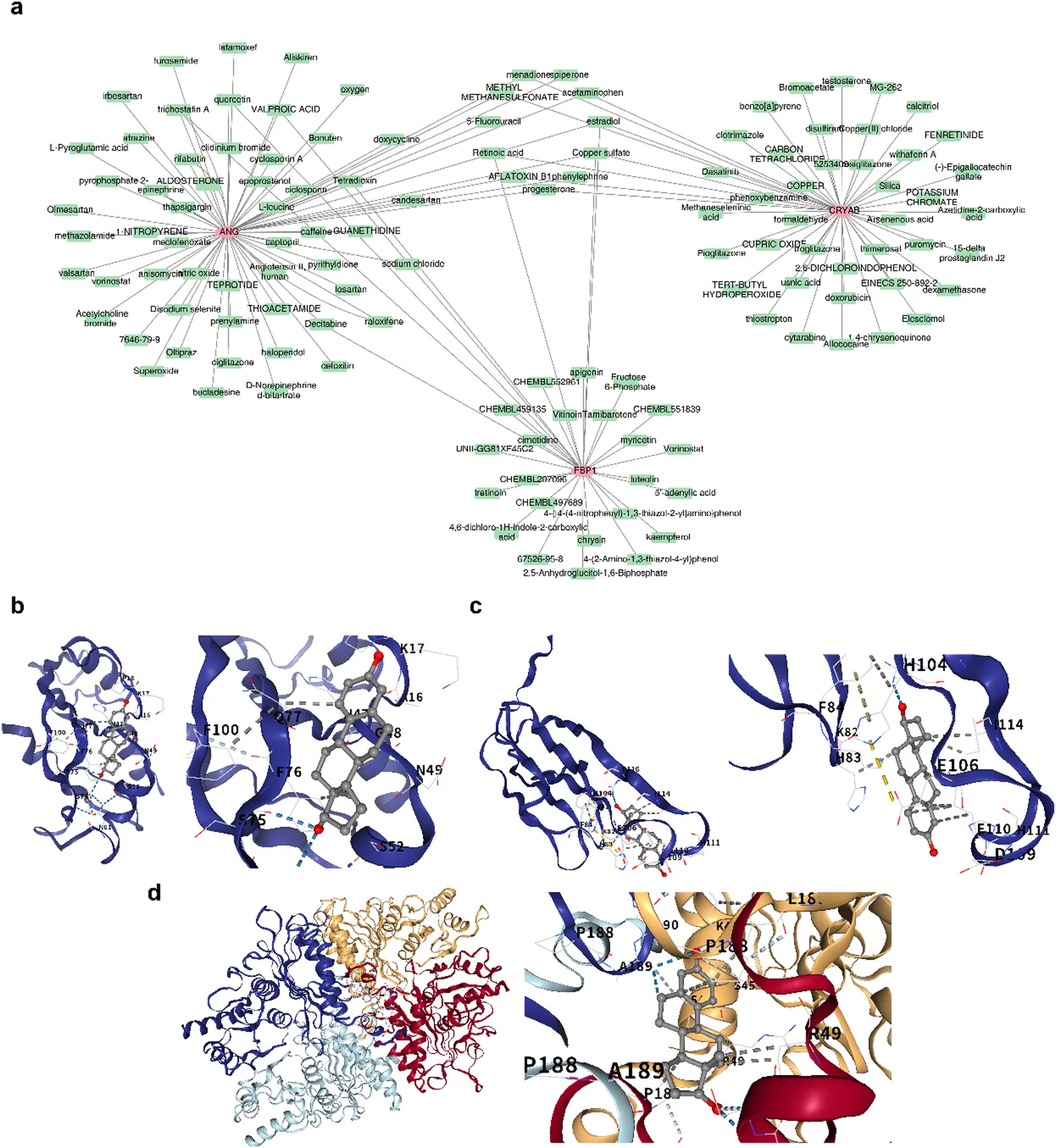
Drug prediction and analysis. (**a**) Biomarker–drug regulatory network. Pink represents biomarkers, green represents predicted drug compounds. (**b**) Molecular docking diagram for ANG and estradiol. The left panel shows the docking diagram of the molecule with the protein, and the right panel provides a close-up view of the docking site, highlighting the binding sites of the small molecule and the gene. (**c**) Molecular docking diagram for CRYAB and estradiol. (**d**) Molecular docking diagram for FBP1 and estradiol.

The drug prediction analysis identified 67 drugs for ANG, 48 drugs for CRYAB, and 31 drugs for FBP1. Among these, estradiol, copper sulfate, and trichostatin A (TSA) were found to interact with all three key genes according to the Degree ranking (Fig. 6a). Specifically, ANG and estradiol share five binding sites, with binding energies of − 6.3, − 5.2, − 5.9, and − 5.3 kcal/mol at sites 1, 2, 3, and 5, respectively, indicating strong binding affinity at these sites (Fig. 6b). CRYAB and estradiol also exhibit five binding sites, with binding energies of − 5.2, − 6.5, − 5.4, and − 5.8 kcal/mol at sites 1, 2, 3, and 5, demonstrating strong binding interactions at these positions (Fig. 6c). For FBP1 and estradiol, five binding sites were identified, with all showing binding energies below − 5 kcal/mol (sites 1–5: − 9.4, − 8.0, − 7.9, − 6.5, − 6.9 kcal/mol) (Fig. 6d). Notably, all five binding sites for FBP1 and estradiol display strong interaction potential, with the binding affinity between FBP1 and estradiol surpassing that of the other two genes.

## Discussion

CRSwNP is a highly prevalent chronic inflammatory disorder affecting the nasal cavity and paranasal sinuses, characterized by severe symptoms and a high recurrence rate. SGs are protective cellular structures that form under stress conditions. These non-membrane-bound cytoplasmic aggregates help cells cope with various environmental challenges. However, the mechanistic relationship between SGs and CRSwNP remains unclear. In this study, transcriptomic data from CRSwNP and SG-related genes from the GEO database were analyzed. Differential expression analysis identified 23 candidate SG-related genes were identified. Combined with LASSO and SVM-RFE machine learning algorithms, ANG, CRYAB, and FBP1 were determined as key genes after ROC validation and expression verification. Further analysis revealed that the three genes are jointly involved in the cell cycle pathway, show a significant positive correlation with type 2 helper T cells, and can interact with drugs such as estradiol. This provides new targets for the diagnosis and treatment of CRSwNP.

ANG (angiogenin) encodes a 14.1 kDa protein from the ribonuclease A superfamily^16^. Its primary function is to promote the proliferation of vascular endothelial cells and angiogenesis, playing a critical role in physiological and pathological processes such as tumorigenesis and wound healing. ANG is essential for the action of vascular endothelial growth factor (VEGF) and fibroblast growth factor-2 (FGF-2)^17^. As a stress-induced, secreted ribonuclease, angiogenin translocates to the cytosol under stress, where it cleaves non-coding transfer RNAs (tRNAs) into fragments, inhibiting ribosome biogenesis and protein synthesis^18^. During cellular homeostasis, ANG promotes ribosomal RNA transcription in the nucleus, and its ribonucleolytic activity is regulated by the ribonuclease inhibitor (RNH1) system^19^. ANG variants have been implicated in neurodegenerative diseases such as Parkinson’s and Alzheimer’s diseases^18^. Increased angiogenesis has been observed in nasal polyps compared to normal nasal mucosa, suggesting ANG’s involvement in the pathogenesis of nasal polyposis^20^. ANG mRNA expression is elevated, and moderate-to-high levels of angiogenin are predominantly localized in the infiltrating inflammatory cells of nasal polyps^21^. This may lead to abnormal proliferation of submucosal neovascularization, providing nutritional support to polyp tissues while exacerbating local edema and inflammatory exudation. These findings suggest a novel therapeutic direction for precision medicine, targeting ANG to suppress polyp growth and alleviate inflammation.

The CRYAB (αB-Crystallin) gene encodes a small heat shock protein (sHSP) that is a member of the molecular chaperone protein family. As a nuclear receptor protein, CRYAB plays a key role in various physiological processes, including human metabolism, immune regulation, and cell differentiation^22^. Mutations in CRYAB, such as CRYAB^R123W^, have been identified as novel causes of hypertrophic cardiomyopathy, linked to pathological NFAT signaling under conditions of pressure overload^23^. CRYAB is also involved in immune cell infiltration and may function as a potential tumor suppressor in certain cancers^24^. Additionally, CRYAB can alleviate cigarette smoke-induced inflammation, apoptosis, and oxidative stress in both normal and diseased human bronchial epithelial cells by inhibiting the PI3K/Akt and NF-κB signaling pathways^25^. Overexpression of CRYAB enhances cell viability and reduces apoptosis, suggesting its potential value in preventing or treating chronic bronchial inflammation, whether in the upper or lower airways, due to the continuous nature of the mucosa. This study represents the first discovery of CRYAB’s involvement in CRSwNP, further establishing its association with the disease.

The FBP1 (Fructose-1,6-bisphosphatase 1) gene is located on chromosome 9 (9q22.32) and encodes a 60 kDa protein composed of 555 amino acids. It is a critical rate-limiting enzyme in glucose metabolism, catalyzing the irreversible hydrolysis of fructose-1,6-bisphosphate into fructose-6-phosphate and inorganic phosphate. This reaction is an important regulatory step in gluconeogenesis and is closely linked to disease development and cellular metabolic reprogramming^26^. FBP1 expression is tightly regulated by hormones, diet, and environmental factors. It is involved in various physiological and pathological conditions, including inherited metabolic disorders, diabetes, hepatic steatosis, and cancer. In cancer, FBP1 acts as a paradoxical gene—it is generally considered a tumor suppressor but can function as an oncogene in certain cancers^26,27^. Downregulation of FBP1 leads to the accumulation of glycolytic intermediates, promoting tumor cell proliferation, stress resistance, and immune evasion. In a murine asthma model, FBP1 was overexpressed, and FBP1 silencing reduced apoptosis and the proportion of cells in the G2/M phase. Overexpression of FBP1 exacerbated oxidative stress by suppressing the nuclear factor erythroid 2-related factor 2 (Nrf2) pathway^28^. Nrf2 is a transcription factor that regulates the cellular response to oxidative and environmental stress. The potential mechanisms of FBP1 in CRSwNP may involve influencing immune cell polarization and function through metabolic regulation, contributing to the metabolic adaptation of the epithelial barrier, and serving as a molecular link between metabolic abnormalities and chronic inflammation. However, there is currently no relevant evidence in this field. This study marks the first identification of FBP1 in CRSwNP.

Subsequent enrichment analysis revealed that the key genes in CRSwNP are regulated through associated pathways. In terms of mechanism exploration, a multi-level regulatory network involving mRNA–miRNA–lncRNA was constructed. GSEA analysis identified the cell cycle pathway as the most significantly shared pathway among the three key genes. The cell cycle pathway refers to the molecular regulatory processes that drive cell division and produce two new daughter cells^29^. The primary function of cell cycle regulation is to ensure accurate and orderly cell division, maintaining stable cell populations and supporting the normal growth, development, and reproduction of organisms^30^. Moreover, the regulation of checkpoints such as the G1/S and G2/M ensures that cell growth and DNA integrity are monitored to prevent genomic instability and tumorigenesis^31,32^. Persistent inflammation and epithelial cell damage, hallmarks of most CRSwNP cases, are linked to cell cycle-related regulation^33^. In the present study, basal cells in CRSwNP patients exhibited excessive proliferation and impaired differentiation^34^. Downregulation of MCM2, a critical protein for DNA replication at the G1/S checkpoint, contributed to the reduced growth potential of epithelial progenitor cells in nasal polyps, thus suppressing the basal cell cycle and promoting abnormal proliferation^35^. This may be a key factor in polyp formation. Additionally, T2 cytokines like IL-4 and IL-13 increase airway epithelial remodeling via an insulin receptor substrate-dependent signaling pathway^36^, disrupting the normal composition and function of nasal mucosal epithelial cells, which accelerates disease progression. The cell cycle pathway thus plays a role in the onset and progression of CRSwNP by responding to inflammatory signals and regulating basal cell proliferation. Further investigation into its molecular mechanisms could provide new directions for precise CRSwNP treatment.

Immune infiltration analysis revealed 23 cell types with significantly differential expression in the training set, predominantly T cells, including activated CD4^+^ T cells, activated CD8^+^ T cells, activated dendritic cells (DCs), CD56^+^ natural killer cells, T follicular helper cells, Th1 cells, Th17 cells, and Th2 cells. Among these, the key genes ANG, CRYAB, and FBP1 exhibited significant positive correlations with Th2 cells. Th2 cells secrete cytokines such as IL-4, IL-13, and IL-5, along with high levels of immunoglobulin E (IgE)^37^. These cytokines activate and recruit numerous inflammatory cells, including eosinophils^38^, basophils, mast cells, goblet cells, M2 macrophages, and B cells, triggering tissue inflammatory responses. During CRSwNP onset, Th2 cells secrete IL-4 and IL-13, which disrupt the nasal mucosal epithelial barrier by inhibiting tight junction proteins and promote excessive basal cell proliferation via cyclin D1 activation^39^. IL-5 specifically recruits eosinophils, which release cytotoxic proteins such as MBP, exacerbating tissue damage and contributing to polyp formation^40^. Additionally, T2 inflammation in CRSwNP is marked by eosinophilic infiltration, elevated IgE, a higher incidence of comorbid asthma and aspirin intolerance, and a significantly increased risk of postoperative recurrence. Th2 cells drive the “inflammation-proliferation-barrier disruption” axis, which governs CRSwNP progression and therapeutic response, making them a central target for disease intervention^41^.

This study conducted a bioinformatics-based exploratory analysis incorporating drug prediction and molecular docking, which preliminarily identified potential predictive targets. Estradiol, a gonadal hormone, has been shown to influence cellular function by interacting with membrane receptors to activate intracellular signaling pathways. Recent studies also highlight that gonadal hormones, including estradiol, function as neurosteroids synthesized locally in the brain, where they can rapidly modulate cognition and neural functions^42^. Estradiol affects T2 inflammatory diseases by modulating Th2-type immune responses^43^, providing a theoretical basis for the targeted hormonal therapy of CRSwNP. Targeting estrogen receptors, particularly ERβ, in the nasal mucosa may inhibit the Th2 inflammatory cascade, tissue remodeling, and polyp recurrence. However, there is insufficient research on the relationship between estradiol and CRS, warranting further investigation. TSA, a histone deacetylase inhibitor, reduces Nox4 expression and angiogenesis by inhibiting the p300 histone acetyltransferase-dependent pathway^44^. TSA inhibits DC maturation by downregulating the NF-κB pathway^45^. In eosinophilic cell lines, TSA-induced transient acetylation does not promote eosinophilic differentiation^46^. TSA also reduces the expression of matrix MMP-2 and MMP-9, and inhibits PI3K and Akt phosphorylation, inducing apoptosis in fibroblast-like synoviocytes under hypoxic conditions in rheumatoid arthritis^47^, suggesting its potential as a novel therapeutic strategy for CRSwNP. However, these findings remain preliminary and require validation through in vitro or in vivo experiments. Future research will aim to further explore the biological functions and therapeutic potential of these key genes and candidate drugs through animal or cellular models.

This study utilized datasets from the GEO database and bioinformatics approaches to identify key genes potentially associated with SGs in CRSwNP treatment. It further explored the biological pathways, immune characteristics, and molecular regulatory networks of these genes, offering valuable insights for future research. However, several limitations exist in the current study. First, the analysis is based solely on publicly available data and lacks prospective clinical sample validation. Second, the sample size of this study is relatively small, which may limit statistical power and pose a risk of overfitting in the nomogram model. Additionally, although the model demonstrated a high AUC value after external validation using the GSE179265 dataset, the calibration analysis was only conducted in the training set and not evaluated in the external validation cohort. As a result, the model may still carry a certain degree of optimistic bias. Moreover, the datasets do not differentiate between eosinophilic (ECRSwNP) and non-eosinophilic (non-ECRSwNP) subtypes, potentially obscuring molecular differences between these subgroups. Finally, the drug predictions and molecular docking results are computationally derived and provide only preliminary exploratory clues for subsequent drug development. Future studies will address these limitations by expanding the sample size and performing multi-center external validation to evaluate the stability and clinical applicability of the key genes and nomogram model. Stratified analyses in populations with well-defined subtypes will also be conducted to explore the differential roles of key genes across different endotypes. Furthermore, cell and animal experiments will be integrated to systematically validate the interactions between the predicted drugs and key genes, as well as their functional mechanisms in CRSwNP, providing more robust experimental evidence for future research.

## Methods

### Data collection

The CRSwNP-related datasets were obtained from the Gene Expression Omnibus (GEO) database. Specifically, the GSE136825 dataset (platform GPL20301) comprises 42 nasal polyp tissue samples and 28 control inferior turbinate tissue samples. To maintain consistency in sample types, paired inferior turbinate tissue samples from CRSwNP patients were excluded from this analysis. The validation dataset, GSE179265 (platform GPL24676), includes 17 nasal polyp tissue samples and 7 control samples. Additionally, 464 SG-related genes were obtained from the Mammalian Stress Granule Proteome (MSGP) database (https://msgp.pt) (Supplementary Table 8).

### Differential expression analysis

DEGs between CRSwNP and control samples in GSE136825 were identified using the ‘DESeq2’ package (v 3.58.1)^48^ for differential expression analysis. DEGs were filtered based on an adjusted P-value < 0.05 and |log_2_ fold change (FC)| > 1. The ‘ggplot2’ package (v 3.5.1)^49^ was used to construct a volcano plot for visualizing these DEGs, and the ‘pheatmap’ package (v 1.0.12)^50^ was employed to generate a heatmap.

### Identification and functional analysis of candidate genes

To identify DEGs associated with SGs, the “VennDiagram” package (v 1.7.3)^51^ was utilized for intersection analysis, with overlapping genes designated as candidate genes. GO (*P* < 0.05) and KEGG^13–15^ (*P* < 0.05) enrichment analyses were subsequently performed using the “clusterProfiler” package (v 4.8.3)^52^, highlighting the top five most significantly enriched terms. Additionally, to explore gene interactions at the protein level, candidate genes were input into the Search Tool for the Retrieval of Interacting Genes (STRING, https://string-db.org/), and the PPI network was visualized using “Cytoscape” software (v 3.10.3, https://igraph.org).

### Identification and validation of key genes

LASSO analysis, a data mining method, was employed using the R package “glmnet” (v 1.7.16)^53^ to perform regression analysis on candidate genes for further screening. In this model, genes with a greater influence on the disease were assigned lower degrees of compression, while those with lesser influence were compressed to zero coefficients. The minimum λ value in the LASSO regression was determined through ten-fold cross-validation, and genes with non-zero regression coefficients were selected, resulting in feature set 1.

SVM–RFE analysis was conducted using the R package “e1071” (v 1.7.16)^53^ to construct an SVM–RFE model for screening DESGRGs. This method identified the gene combination corresponding to the lowest error rate as the feature genes for SVM–RFE. K-fold cross-validation (k = 10) was applied, with the feature set divided into two halves in each round until fewer than 100 features remained. Feature gene set 2 was ultimately obtained through SVM–RFE screening. ROC curves were constructed using the “pROC” package (v 1.18.0)^54^, and the 95% confidence intervals for the AUC values of the LASSO and SVM–RFE models were calculated using the Bootstrap resampling method (repeated 1,000 times).

Next, the ‘VennDiagram’ package (v 1.7.3) was used to find the intersection of feature gene set 1 and feature gene set 2, yielding the final feature genes. To assess the ability of these feature genes to distinguish CRSwNP tissue samples from control tissue samples, receiver operating characteristic (ROC) analysis was performed using the “pROC” package (v 1.18.0)^54^ on feature genes in both the training and validation sets. AUC values were calculated, and feature genes with area under the curve (AUC) values greater than 0.7 and not equal to 1 in both sets were selected as candidate key genes.

To examine the expression of candidate key genes in the training and validation sets, the Wilcoxon rank-sum test was applied to compare expression levels between CRSwNP and control samples (*P* < 0.05). Genes with differential expression across samples and consistent trends in both datasets were selected as key genes.

### Construction of nomogram and subcellular localization

To evaluate the predictive value of key genes for CRSwNP, a nomogram was constructed using the R package “rms” (v 6.8.1)^55^ based on the CRSwNP and control tissue samples from the training set GSE136825. The nomogram assigns scores to each key gene according to its expression level, sums the scores of all key genes to obtain a total score, and estimates the probability of CRSwNP based on this total score—the higher the score, the greater the likelihood of CRSwNP.

To assess the prediction accuracy of the nomogram:

1. DCA was performed using the R package “ggDCA” (v 1.2, doi:https://doi.org/10.1177/0272989X06295361), and the corresponding DCA curve was plotted.
2. A calibration curve for the nomogram was generated using the R package “rms” (v 6.8.1).

To evaluate the prediction stability, ROC analysis was performed on the nomogram using the “pROC” package (v 1.18.0) in both the training set and the validation set, with the threshold set at AUC > 0.7.

The subcellular localization of all key genes was predicted using the mRNALocater database (http://bio-bigdata.cn/mRNALocater/).

### Gene set enrichment analysis (GSEA)

To explore the functional pathways associated with key genes in CRSwNP, GSEA was performed. The reference gene set ‘c2.cp.all.v2022.1.Hs.symbols.gmt’ was selected from the Molecular Signatures Database (MSigDB). First, Spearman correlation analyses were conducted between each key gene and all other genes in the training set using the “psych” package (v 2.4.3)^56^, generating correlation coefficients. Genes were then sorted in descending order based on these coefficients. The sorted data were used to conduct GSEA (|normalized enrichment score (NES)| > 1, FDR < 0.25, *P* < 0.05) using the “clusterProfiler” package (v 4.8.3)^52^. The top 5 upregulated and top 5 downregulated pathways for each of the 3 key genes were selected.

### Establishment of Molecular Regulatory Networks and RBP Networks

To further explore the regulatory mechanisms of key genes in CRSwNP, miRNAs targeting key genes were predicted using three databases: DIANA-microT (http://mirtoolsgallery.tech/mirtoolsgallery/node/1084), miRanda (http://www.microrna.org/), and TargetScan (https://www.targetscan.org/). The “ggVenndiagram” package was used to identify the intersecting miRNAs predicted by these databases (note: the original text mentions “5 databases,” but only 3 are listed, which may be a typo). These intersecting miRNAs were further analyzed.

For lncRNA–miRNA interaction data, key lncRNAs upstream of the intersecting miRNAs were obtained from the starBase database (https://starbase.sysu.edu.cn/), with lncRNAs filtered based on clipExpNum > 4. The Cytoscape software (v 3.10.3)^57^ was used to construct the molecular regulatory network of key genes. Finally, to explore RNA-binding proteins (RBPs) associated with key genes, the ENCORI database (https://rnasysu.com/encori/) was used to predict potential RBPs interacting with key genes (thresholds adjusted to clusterNum ≥ 1 and clipExpNum ≥ 1), and the Cytoscape software was employed to build the key gene–RBP regulatory network.

### Immune infiltration analysis

To investigate immune infiltration patterns across different samples, the R package “GSVA”^58^ was utilized to calculate the infiltration percentages of 28 immune cell types in each sample from both the CRSwNP and control groups. The R package “ggplot2” was then used to generate heatmaps illustrating the abundance and proportion of immune cell infiltration in both groups. The Wilcoxon test was subsequently performed to assess differences in the infiltration levels of these 28 immune cell types between the CRSwNP and control groups (*P* < 0.05), with results visualized using “ggplot2” (v 3.5.1)^49^. Additionally, Spearman correlation analysis was conducted to examine the associations between differentially infiltrated immune cells and key genes, as well as among the immune cells themselves (|correlation coefficient (cor)| > 0.3, *P* < 0.05).

### Drug prediction and molecular docking for key genes

To identify potential drugs for CRSwNP, drug compound prediction was carried out for the key genes using the Drug Signatures Database (DsigDB, http://dsigdb.tanlab.org/). Drug-compound-biomarker interactions were predicted, and the interaction network was visualized using Cytoscape software. Molecular docking analysis was then performed: the protein crystal structures of key genes were obtained from the PDB database (https://www.rcsb.org/), and the 3D structures of active compounds were sourced from the PubChem database (https://pubchem.ncbi.nlm.nih.gov/). Molecular docking was conducted using the cbDOCK2 online platform (https://cadd.labshare.cn/cb-dock2/php/index.php) to assess the binding energy between active compounds and key genes. The docking mode with the lowest binding energy was selected as the optimal binding pose, and the results were visually displayed.

### **Statistical analysis**

All bioinformatics analyses were performed using R programming language (v 4.3.1). Differences between the two groups were evaluated using the Wilcoxon test, with a significance threshold set at *P* < 0.05.

## Supplementary Information

Below is the link to the electronic supplementary material.

Supplementary Material 1

Supplementary Material 2

Supplementary Material 3

Supplementary Material 4

Supplementary Material 5

Supplementary Material 6

Supplementary Material 7

Supplementary Material 8

## Data Availability

The datasets analysed during the current study are available in the Gene Expression Omnibus database repository, [http://www.ncbi.nlm.nih.gov/geo/], including GSE136825 dataset and GSE136825 dataset.
